# Research on Service-Learning in Physical Activity and Sport: Where We Have Been, Where We Are, Where We Are Going

**DOI:** 10.3390/ijerph19116362

**Published:** 2022-05-24

**Authors:** Xavier Francisco-Garcés, Celina Salvador-Garcia, María Maravé-Vivas, Oscar Chiva-Bartoll, María Luisa Santos-Pastor

**Affiliations:** 1 Facultad de Ciencias de la Actividad Física y el Deporte, Universitat de València, 46010 Valencia, Spain; xafran2@alumni.uv.es; 2 Department of Education and Didactics of Specific Subjects, Universitat Jaume I, 12071 Castellón, Spain; salvadoc@uji.es (C.S.-G.); ochiva@uji.es (O.C.-B.); 3 Department Physical Education, Sport & Human Movement, Universidad Autónoma de Madrid, 28049 Madrid, Spain; marisa.santos@uam.es

**Keywords:** service-learning, review, physical activity, higher education, research

## Abstract

Higher education is under constant transformation through the use of new pedagogical models such as university service-learning (SL). Indeed, there has been an exponential uptake of university SL, among others, in the field of physical activity and sport (PAS) along with research examining these practices. However, these initiatives highlight the need to improve the quality of research in this field. This paper presents a systematic review focused on how research in this arena has been carried out, examining the following topics: paradigm, methods, instruments, discipline, limitations, and further research. A total of 45 articles met the inclusion criteria. The results show that qualitative and mixed methods have experienced an increasing progression. The most recurrent instruments have been questionnaires, reflective diaries, and interviews. According to the studies in the sample, the limitations point to research designs and some difficulties that underlie the pedagogical model itself. Finally, further research calls for longitudinal studies and to deepen the reflective process. This review identifies some weaknesses and strengths of research in university SL in PAS that aspire to inform and improve future investigations in this field.

## 1. Introduction

The exponential uptake of service-learning (SL) in the field of physical activity and sport (PAS) has been well documented by a series of reviews published in recent years [1,2,3,4,5]. This comes as a consequence of two factors: (1) the promotion of active and participatory methodologies in higher education, stemming from the demand for higher institutions to develop university social responsibility; and (2) the beneficial effects of SL on students’ development.

There is a need to find ways to promote integral education since higher education students should be able to respond to the needs of today’s society. To address this need, applying active teaching methodologies aligned with sustainable development goals (SDG) seems to be a reasonable option [6,7]. In addition, Meztler [8] highlights the need for PAS teachers to leave behind traditional teaching methods and bet on new approaches that meet the requirements of today’s society. In this line, active and participatory methodologies seem to be adequate due to their intrinsic characteristics.

Specifically, SL represents a pedagogical approach with the potential to be instrumental to foster such a transformation. In fact, it promotes the development of university social responsibility [9], because SL is an educational experience framed in a subject in which the students provide a service aimed at benefitting the community. Concomitantly, it comes with a number of benefits for students such as a greater understanding of the content of the subject, or personal values and civic responsibility promotion [10]. As a consequence, a range of universities have embraced and started to apply SL in order to foster civic objectives [11].

The literature highlights not only the positive effects that SL generates on university students but also how this pedagogical approach meets current professional needs [12]. Among its benefits, previous literature points to the development of positive attitudes towards special educational needs, the development of a deeper understanding of inclusion and the improvement of confidence in students’ ability to manage inclusive educational experiences [13,14]; the development of empathy [15] or the increase in civic engagement [16]. SL generates a type of interaction and participation that allows students to better understand themselves as well as the other agents involved in the learning process while promoting reflection, communication strategies, and social skills [17,18]. Nevertheless, besides the aforementioned benefits, it is necessary to point out that the research and application of university SL in PAS also come with a series of limitations or risks that should be examined and shared, (i.e., biased viewpoints, power relationships that are not changed, ethnocentric approaches). These critical ideas, in fact, are desirable to inform researchers and scholars focused on this topic.

When research is designed, it is necessary to carefully consider cardinal factors such as the paradigm through which the investigation is to be approached. Khun [19] defines paradigms as universally recognized scientific processes that guide a scientific community to solve problems over a period of time. According to Ricoy [20], the positivist paradigm is quantitative, empirical-analytical, rationalist, and systemic. Thus, it involves research aiming to test a hypothesis through statistics or determine the parameters of a given variable using numerical expressions. For its part, the interpretive paradigm focuses on analyzing the meanings of human interactions, considering the power of the context. This type of research aims to provide explanations since it attempts to interpret and understand human behavior according to the meanings of the individuals involved in a specific scenario [21]. Finally, the sociocritical paradigm stems from social criticism and self-reflection. Particularly, it aspires to describe and understand social relations through the participation of individuals as well as the subsequent social transformation [22]. To identify the paradigm followed in a study, it is necessary to analyze different items. For instance, within the educational field, Schuster et al. [21] present a classification of these three types of paradigms according to the following components of the research process: research problem, design, participants, data collection techniques, data analysis and interpretation, and research assessment (Table 1).

Methodological rigor and coherence between research and SL application is a relevant aspect that has not been specifically addressed in existing reviews since they have focused on other issues. For example, Cervantes and Meaney [23] carried out a review to examine the theoretical frameworks used and the impact of SL on students and the social community. They also presented some suggestions for future practice. Another review in the field of PAS with young people was carried out by Carson and Raguse [1]. They classified the results into three different categories: an overview of the research, a summary of the program, and salient strategies for its application. Both of these reviews evinced the significant increase in research examining SL and shared a myriad of high-impact programs that could be replicated later on. Salam et al. [24] carried out another comprehensive systematic review of articles confined from 2000 to 2018. These authors examined the academic disciplines adopting SL, the emerging issues for SL integration, the limitations of SL frameworks, and its potential outcomes.

Recent reviews of SL in PAS have become more specific. Chiva-Bartoll et al. [3] described the different methodological approaches used in the studies of their sample, the effects that SL had on both service providers and service receivers, as well as the duration and intensity of each program or intervention. In other words, they examined the intricacies of the SL programs, mainly from a pedagogical lens. Pérez-Ordás et al. [5], on their part, analyzed the implementation of SL programs in physical education teacher education. The aim of their review was to examine the effects of SL on the professional, social and personal skills of the students and its impact on the social community. In addition, they analyzed the effectiveness and quality of the programs.

Other reviews approaching SL in PAS have analyzed a specific context or group of receivers. For example, Ruiz-Montero et al. [4] centered their review on health care promotion programs for older adults; while Case et al. [2] carried out a meta-analysis in order to analyze the effects of SL programs applied to people with functional diversity. Again, these papers focus on the programs and their effects on the participants, thus sidelining research specifications.

The contributions of all these reviews on SL in the field of PAS are the starting point from which new lines of research emerge. Particularly, these reviews highlight the need to take stock specifically of the research approaches that have been implemented. Consequently, there is a call for reviews addressing this gap, which still remains unexplored in this field of study. That is to say, there is a need to carry out a review focusing on the research processes and designs used by scholars in order to gather data and inform future investigations.

## 2. Materials and Methods

### 2.1. Objective

The objective of this paper is to identify and analyze the scientific literature on SL in the field of PAS in the last five years since there is an important increase in the amount of research published in recent years in this field. Specifically, it aims at delving into the research paradigms used, methods applied, the data collection techniques chosen, as well as the limitations and future research ideas mentioned in these studies in order to provide the scientific community with useful ideas that may inform future applications and research.

Bearing in mind this objective, the research questions to be answered are: how many studies have been published on SL in PAS in the last five years? Where have these studies been carried out? What paradigms of study are used? What are the research methods that these studies employ? What are the data collection techniques used? What are the limitations elicited in these studies? What are the future implications that these papers propose? This combination of questions aspires to clarify what aspects should be considered when starting an investigation on SL in PAS.

### 2.2. Search Strategy

To carry out this review, we followed the guidelines proposed by Moher et al. [25]. The search gathered those studies that have been published in both English and Spanish since these were the languages mastered by the research team. In addition, the search was conducted in two of the most relevant scientific databases in the educational field, namely Web of Science and Scopus (Elsevier). The same descriptors were used in both databases. In the first field of descriptors, we established “service-learning” OR “service learning” OR “experiential education” OR “experiential learning” for the title, keywords, or abstract. In the second field, we added the operator AND to add the following terms: “physical education” OR “activity” OR “physical activity” OR “sport education”.

Bearing in mind the aforementioned objectives, a set of inclusion and exclusion criteria was established. These let us collect only those papers that were instrumental for the purposes of our study. The criteria applied were:

1. We narrowed down the search by considering only those studies written in English or Spanish since these are the languages mastered by the research team.
2. We established the publication interval to be between January 2016 to March 2021, bearing in mind the recent increase in the number of papers revolving around SL in the area of PAS [24].
3. We filtered the search considering the document type since this review was focused specifically on gathering research papers.
4. We filtered the search considering the area of knowledge; thus, we searched for papers published on the topic of social sciences and linked to teaching and education.
5. We excluded all the papers that did not deal with SL in PAS or were not linked to education.

**Figure 1:**
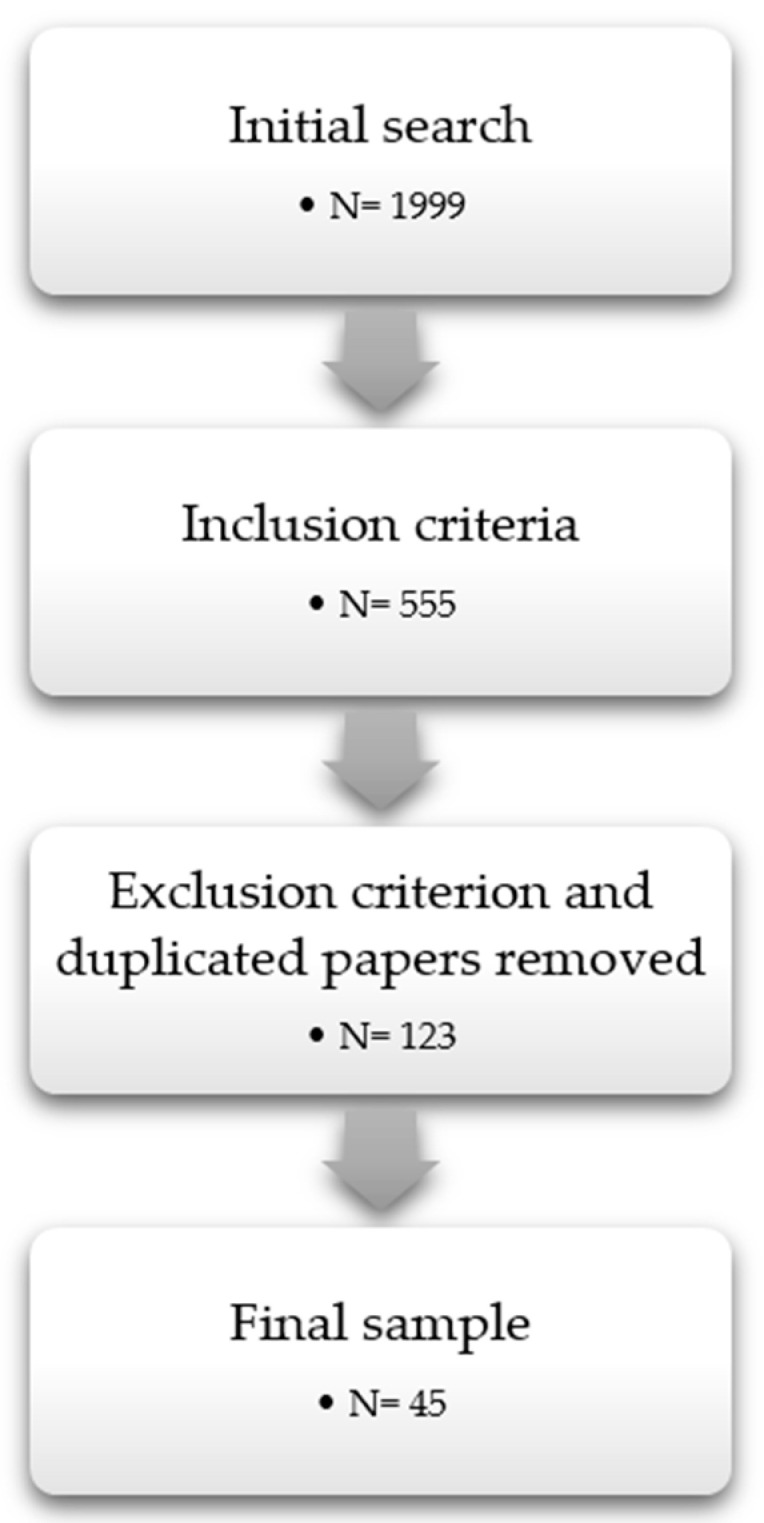
Search protocol.

We took as a starting point PRISMA guidelines for systematic reviews [25]. Thus, the first step consisted of the identification stage [25]. This search yielded a total of 1999 publications when the search equation was applied. Next, we moved to the screening step [25]. Therefore, we applied the aforementioned inclusion criteria by examining the title of the papers gathered. At this point, the sample was narrowed down to 555. After that, duplicated papers and texts that were not within the scope of this review were removed. At this point, the sample diminished to 123 publications. The research team read the abstract and the keywords, taking into account the research objective established. In case of doubt, the full paper was read at this stage to make sure it was relevant for the purposes of this review. Subsequently, the research team carried out an exhaustive reading of all the papers of the sample in order to extract the content to be used in this study. Two independent researchers extracted and selected these data. The discrepancies between them were contrasted and discussed with a third researcher until a consensus was reached. Finally, once the content of the papers was extracted, only 45 of them provided us with sufficient information to be part of the final sample (Figure 1).

### 2.3. Classification Criteria

Table A1 shows all the studies included in the final sample. These are classified considering the following items:

- Publication date and geographical distribution (country).
- Research paradigm, (i.e., positivist, interpretative or sociocritic).
- Research method, (i.e., quantitative, qualitative, or mixed-methods).
- Research instruments.
- Limitations of the study, according to the ideas expressed in each paper.
- Future research, according to the ideas expressed in each paper.

## 3. Results

This section presents the results organized in different subsections according to the aforementioned items.

### 3.1. Publication Date and Geographical Distribution

**Figure 2:**
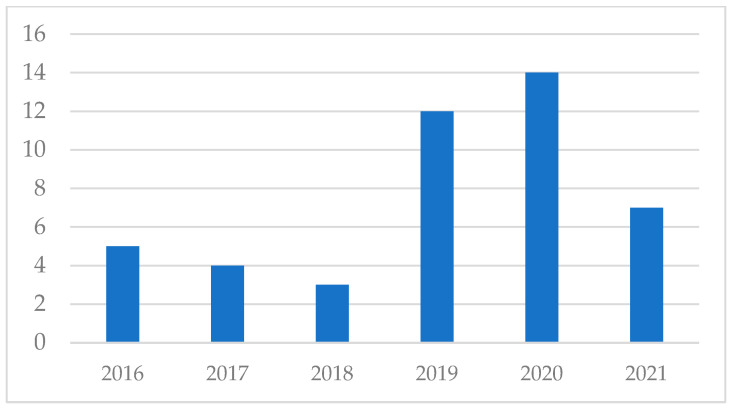
Amount of papers by year of publication.

First, regarding the publication date of the papers of the sample, results show a substantial increasing tendency between 2016 and 2021, which is particularly notorious in the last three years. In fact, the papers published in 2019 and 2020 (*n* = 26) represent 56.52% of the sample. In addition, this trend seems to continue in the year 2021, as in just three months, seven papers have been published, which is the same number of papers gathered in the whole year 2017 or 2018. Figure 2 shows the number of publications classified by date of publication.

**Figure 3:**
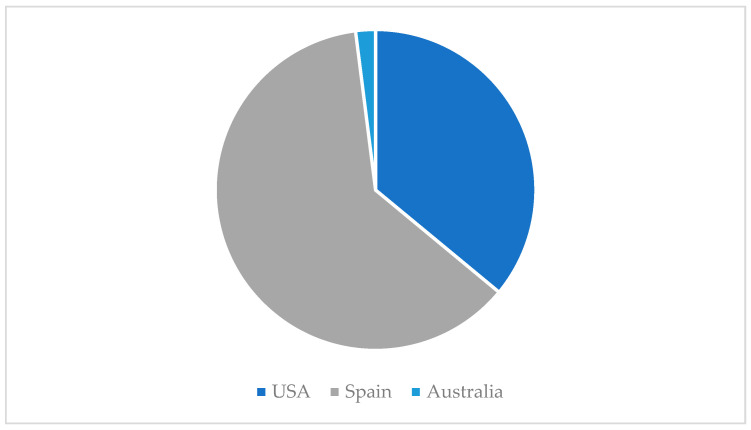
Geographical distribution.

Second, considering the country where the research teams were settled, results evince a clear hegemony of Spain (62%) followed by the United States of America (36%). In fact, only one paper of the sample was carried out in Australia (2%) [26] (Figure 3).

### 3.2. Paradigms

**Figure 4:**
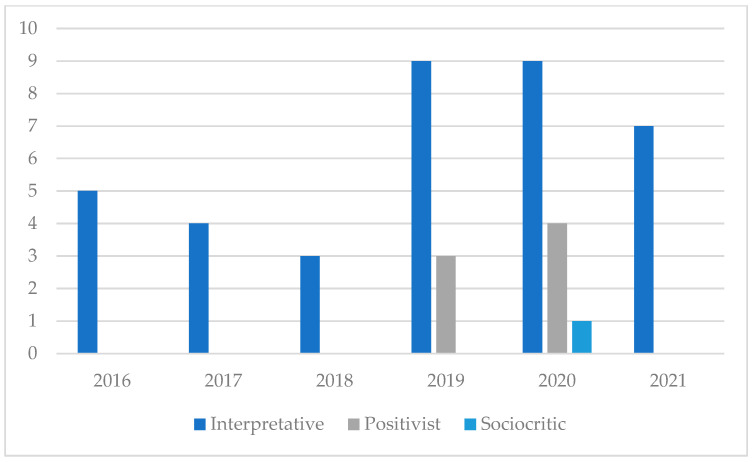
Research paradigm by publication date.

This subsection presents the results of the review regarding the research paradigms of the papers in the sample. In order to carry out this classification, we followed the guidelines established by Shuster et al. [21]. It is worthy of note, though, mentioning that this classification may be subject to different interpretations. Our findings suggest that most of the papers adopt an interpretative paradigm. This is the case, for example, of An’s [27] study, whose methods section specifically alludes to this approach in order to describe and understand the participants’ experiences from an interpretative perspective. The positivist paradigm is the second most frequent. In these cases, the research design, the instruments used, and the quantitative methodology attempt to measure the effects of SL [28,29,30,31]. Finally, a single paper was classified in the third group [32]. Its research paradigm was considered to be sociocritical because in the introduction section and the future implications the authors highlight the interests and needs of the participants from a political perspective. However, this study could have been placed within the interpretative approach too. Figure 4 presents the research paradigms of the papers by year of publication.

### 3.3. Methods

**Figure 5:**
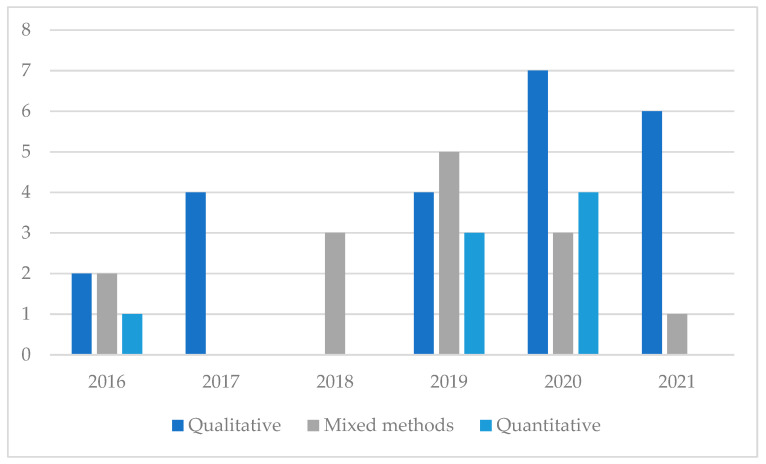
Research method by year of publication.

The next analysis consisted of examining the research method used in the papers conforming to the sample. In this respect, qualitative approaches were widely used. In fact, 23 papers (51.11% of the sample) followed this type of approach. The other half was divided into 30.43% of papers using mixed methods and 19.57% following a quantitative approach. Regarding the tendency by year of publication, the qualitative approach has been increasing its relevance recently. However, mixed methods are also gathering momentum (Figure 5).

### 3.4. Data Gathering

This section focuses on the research instruments used. In this sense, on the one hand, qualitative studies, which tended to follow an interpretative approach, gathered data through reflective diaries or narratives [27,33,34,35,36,37]. Indeed, these are the most recurrent instruments considering the whole sample. Other frequently used instruments in qualitative studies were conversational techniques, among which we may find semi-structured interviews [32,36], or focus groups [38,39]. Additional instruments that were not so widespread among qualitative investigations were visual methods, such as recordings or video diaries [27,40,41,42].

On the other hand, moving now to the quantitative investigations or the quantitative part of those using mixed methods, a very recurrent research instrument was questionnaires [28,30,43,44,45,46,47,48,49,50]. The validated questionnaires used were the *Entrepeneurship Competency Scale* [51], the *Subjective Happiness Scale* [28], and the *Multidimensional Attitudes Scale Toward Persons With Disabilities questionnaire* [29], among others. Furthermore, some specific instruments were used on a few occasions. These ranged from the Movement Assessment Battery for Children-2 [52] or triaxial accelerometers [31] to the FITNESSGRAM test [53], which were used to analyze the effects of SL on service receivers.

**Figure 6:**
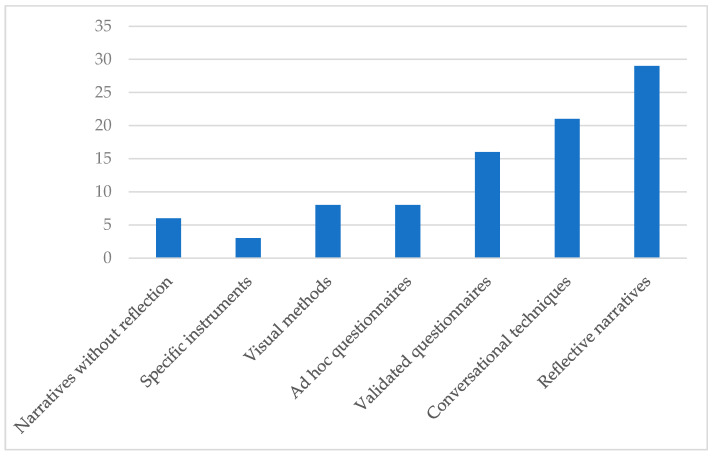
Frequency of use among the research instruments.

Figure 6 shows the number of times each instrument has been used in the papers conforming to the sample.

### 3.5. Reported Limitations

Each paper of the sample reports a series of limitations of the study itself. Therefore, this section presents such limitations, which have been divided into, on the one hand, limitations related to the research and, on the other, limitations linked to the SL implementation.

First, regarding the research limitations, we have found the following:

- Research design. Some papers express that their investigations were short, thus limiting their understanding of the long-term effects of SL [26,33,34,46].
- Sample. Convenience samples or self-selection participants were used on several occasions [28,42,44,48,54,55].
- Social desirability bias. This type of bias was identified in the answers of some research participants [26,33,42,56].
- Results generalizability. A number of studies recognize that the sample size does not allow for generalizing the results obtained. In fact, research teams accept this limitation very explicitly [30,31,33,40,44,45,48,51,52,53,54,57,58,59,60].
- Instruments. The format of some research instruments could have hindered a profound understanding of the object of study, as it was mentioned in the paper by Bush et al. [33]. Likewise, some constructs, such as the *Effective personality,* may be considerably difficult to measure [44]. Finally, additional variables could have influenced the results, but the instruments were not focused on them [57].

Moving now to the limitations related to the SL implementation, there are a number of issues that were reported in the papers of the sample. Following Rubio and Escofet [61], we established the following basic categories of limitations:

1. Basic dynamisms. This type of limitation may encompass, educators’ limited training and experience regarding SL [45,46], limited time of engagement in the program on the part of participants [27,50], and difficulties when adjusting the service to the specific needs of the social community [41], or the intrinsic difficulties coming with the features of the people receiving the service [27].
2. Pedagogical dynamisms. Some participants reported that SL entailed a complex and challenging process through their reflections. This may be perceived in the studies as giving voice to the participant students’. For instance, undergraduates could feel frustration, confusion, changes in their mood, or mental disequilibrium [37,62].
3. Organizational dynamisms. On many occasions, time was considered to be a limiting factor, since programs tended to be short-term [26,33,34,45,50,63] Furthermore, the context where the program was developed was perceived as a limitation in some cases [27,55]. Another limitation in this sense, was the management of the program because SL comes with great effort and time devoted to designing, planning, organizing, etc. [50].

### 3.6. Future Research and Implications

The last section of the results focuses on the future research and implications mentioned in the papers conforming to the sample. This section has been divided into the following three blocks:

(a) More deepening. Some papers report that there is a need to continue delving into the viewpoints, perceptions, experiences, etc., of all people involved in the SL programs in PAS. This included not only students but also the rest of the agents that are part of these programs [27,34,38,42,43,45,56].
(b) Improvement of the program. According to some authors, SL programs in PAS should be longer and/or provide the service more frequently. This might enhance the program and thus, their effect on students, service receivers, and society could be greater, since there would be more interaction and shared participation [26,28,34,44,51,52,57,60].
(c) Design enhancement. Some papers advocate for using qualitative methodologies in order to delve deeper or even complement other types of research [53,57]. In addition, other possibilities could be adopting longitudinal designs [26,34,44,51,52,57,60] or involving other areas, degrees, or subjects in the SL programs [57,64].

## 4. Discussion

This systematic review has gathered a compendium of publications examining SL in PAS with the aim of identifying and analyzing how research has been carried out in this field. Regarding the results obtained, investigations revolving around SL in PAS are increasing. This might come as a consequence of the fact that active and participative methodologies are reaching a new height in higher education to respond to university social responsibility, and SL is considered to be an optimal approach in this sense [9,11,16,17,18,30].

Focusing on the geographical distribution of the sample, the USA and Spain stand out. This result is in accordance with previous reviews [3]; however, we could perceive that the Spanish production took off significantly in 2019, and it is still following a rising tendency.

Another aspect this review has examined is the epistemological positioning when carrying out research. In this review, the studies have been classified considering the three most recurrent paradigms in educational research [21]. The results show a clear predominance of the interpretive paradigm. This could come as a result of many papers focusing on the analysis of the meanings of human interactions when applying SL. According to Schuster et al. [21], this paradigm attempts to interpret and understand human behavior by considering the meanings of each participant within the educational scene. Since many of the investigations of the sample analyze learning in terms of values, skills, or attitudes; the interpretative paradigm emerges as a coherent option in these cases. These studies tended to focus on the perception, reflection, and experiences of the people who carried out the service. However, there were some examples that considered service receivers’ viewpoints too.

In accordance with the aforementioned ideas, the results regarding the methodological approach come with no surprise, since they reveal a notorious tendency towards the use of qualitative approaches and mixed methods. On the one hand, these results align with previous reviews on SL [5,23,65]. On the other hand, selecting mixed or qualitative approaches could have been subject to the phenomena or variables to be analyzed. These methodological approaches allow for a holistic and in-depth analysis of an object of study in its own context and aspire to understand the complexity of social phenomena based on the perspective of its participants [66,67]. Although, to a lesser extent, quantitative studies have also been found. Almost all of these have been classified under the positivist paradigm and have used validated questionnaires as instruments.

In relation to the data collection instruments, qualitative studies tended to rely on reflective narratives or conversational techniques such as interviews, in line with the findings of Pérez-Ordás et al. [5]. Nevertheless, we also identified visual methods as an additional procedure to be added to the aforementioned ones. Visual methods are associated with the field of sociology and anthropology, and their use is still quite limited in education [68]. In spite of this, there has been a surge in their use in educational studies [69]. Although this data collection option is usually linked to interpretative qualitative research, it may also empower the participants while helping to shed light on critical elements of the practice that could be complex to express through words [70].

Another topic examined in this review dealt with the limitations of SL research in the PAS arena. In this sense, on many occasions, research teams pointed out that their results could not be generalized. Regarding the number of participants, many studies relied on small samples, and researchers explicitly accept this limitation [30,31,33,40,44,45,48,51,53,54,57,58,59,60]. Albeit the issue of generalization should be taken into account, it must be interpreted with caution. As we mentioned before, many of the studies adopt an interpretative approach. This means that they aspire to understand and perceive a concrete reality, not to obtain overarching laws or generalizable principles. Therefore, this type of study is not expected to find a generalizable result. This is a complex issue that could be influenced by the nomotechnic generalization of the results that quantitative approaches aim to foster. However, qualitative researchers can follow robust and accurate approaches to their investigations. For instance, they could consider issues such as transferability, understood as the possibility of extending the findings to other populations in qualitative studies [71].

Another limitation related to the participants is the composition of the sample, as many studies used self-selection or convenience sampling. These remarks are useful for future researchers since they will be able to take them into consideration when designing their studies. Furthermore, some authors such as Chiva-Bartoll et al. [28] share possible solutions in this sense, such as conducting randomized trials to reinforce validity.

Furthermore, one of the most significant outcomes of this review consists of the gathering of possibilities for future investigations on SL in PAS. Despite the differing nature of the sample analyzed, there is a shared call to give a voice to all the agents involved in SL. In this line, there are studies highlighting the need to continue reflecting on this aspect [72] and specifically to reflect with the participant community [49]. It is true that some elements to encourage shared reflective processes are starting to be incorporated by inquiring social entities [41] and service receivers [73]. Nevertheless, most of the investigations remain to be focused solely on higher education students and only refer to the promising role that entities and service receivers may have as lines of future research. Perhaps this type of scientific production could be more prolific and relevant in other areas of knowledge, such as social education [74]. Regardless of this, if researchers endeavor (1) to be faithful to the transformative nature of SL and (2) to deepen into the knowledge of SL, investigations should start to consider and give voice to all the social community through co-production research [75]. Specifically, co-production research aspires to move from the idea of “research about” to that of “research with”. This means that knowledge creation should not only rely on the researchers’ and higher education students’ viewpoints. Instead, shared participation processes should be promoted along the different stages that research entails. From this perspective, all agents involved in an investigation are able to participate, for example, in research design and result dissemination. Therefore, social transference and the impact of future educational practices are expected to be improved [76]

Another future research implication repeated in a number of papers highlighted the need to carry out longitudinal studies [26,33,34,46]. The literature in the field of SL in PAS has stressed this idea for a long time [3,4,77,78,79]. Nevertheless, according to our findings, this request is yet to be responded. These designs may be instrumental to identify potential threats or needs from a long-term perspective thus, providing fresh ideas on how to optimize SL programs [80].

SL can be an adequate pedagogical approach since it allows a transformative approach to social justice in higher education while offering valuable services to the local community. Nevertheless, it also has its drawbacks and disadvantages [81]. Among these, we may find the complexity of applying SL projects out of the class and in real contexts [82] or the length and magnitude of these projects [51]. This may be due to the fact that SL tends to be applied in connection to a single subject thus being conditioned by students’ previous training, and time to properly reflect, understand, and discuss with all agents involved [83]. Consequently, with this review, we aspired to spotlight how research on SL in PAS may be improved to optimize the application of this pedagogical approach [84].

## 5. Conclusions

The objective of this systematic review was to analyze “how” SL in PAS has been investigated in order to share guidelines to blueprint new initiatives in this arena. This is relevant since SL research in PAS continues to grow, evincing the transformation undergone by higher education institutions to foster quality education. Consequently, the results obtained in this review evince that SL should gather essential elements to be considered as a transformative, activist, and intercontextual pedagogical method [85]. Therefore, it is critical to identify the challenges and complexities that may restrict SL in PAS. Our results have provided educators with ideas and possibilities to enhance the implementation of this pedagogical method while spurring researchers to increase the quality of their investigations.

In short, this work highlights the main strengths and weaknesses found in SL studies in the field of PAS, aiming at contributing to better applications and research of this pedagogical model. This review does not come without certain limitations such as the languages used in the search, the specific publishing dates of the articles reviewed, or the fact that its scope was limited to the review of specific literature, which may have biased some of the results obtained. In any case, the findings of this review may make a significant contribution to the overall body of knowledge, reinforcing the case to continue using SL and investigating its effects by pulling together the broad and disparate strands of the literature in the field of SL in PAS.

## Figures and Tables

**Table 1:** Methodological derivations of research paradigms.

	Positivist	Interpretative	Sociocritic
**Research problem**	Theoretical	Perceptions and feelings	Experiences
**Design**	Structured	Open and flexible	Didactic
**Participants**	Procedures	Not specified	Interests and needs of the individuals
**Data collection techniques**	Reliable instruments	Qualitative techniques	Personal communication
**Data analysis and interpretation**	Statistical techniques	Reduction, exposition, and conclusion	Group’s participation in the analyses
**Research assessment**	Internal and external assessment, reliability, and objectivity	Credibility, transferability, dependability, and confirmability	Consensual validity

Note. Adapted from Schuster et al. [21].

## Data Availability

Not applicable.

## Appendix A

**Table A1:** Studies included in the final sample.

Authors, Year and Country	Paradigm	Method	Instruments	Limitations	Future Research
Bush, K. A., Edwards, M. B., Jones, G. J., Hook, J. L., and Armstrong, M. L (2016) [33]. USA	Interpretative	Qualitative	Ad hoc questionnaire Reflective narratives	Sample Generalizability Instruments Bias SL implementation Design	Enhance design Deepening more
Peralta, L. R., O’Connor, D., Cotton, W. G., and Bennie, A. (2016) [26]. Australia	Interpretative	Mixed methods	Reflective narratives Validated questionnaire Conversational techniques	SL implementation Design Bias	Deepening more Improve the program
Santiago, J. A., Lee, J., and Roper, E. A. (2016) USA [54].	Interpretative	Quantitative	Validated questionnaires	Sample Generalizability Bias Instruments Design	Deepening more Enhance design
Gil-Gómez, J., Moliner-García, O., Chiva-Bartoll, Ó., and García López, R. (2016) [45]. Spain	Interpretative	Mixed methods	Validated questionnaire Reflective narratives	Sample SL implementation Generalizability	Deepening more
Martin, T., Warner, S., and Das, B. (2016) [56]. USA	Interpretative	Qualitative	Narratives without reflection	Bias Generalizability	Enhance design Deepening more
Ward, S., Pellett, H. H., and Perez, M. I. (2017) [37]. USA	Interpretative	Qualitative	Conversational techniques Visual methods	Bias SL implementation	Deepening more
Webster, C.A., Nesbitt, D., Lee, H., and Egan, C. (2017) [39]. USA	Interpretative	Qualitative	Conversational technique Reflective narratives	Design SL implementation	Deepening more
Whitley, M. A., Farrell, K., Maisonet, C., and Hoffer, A. (2017) [42]. USA	Interpretative	Qualitative	Reflective narratives Visual methods	Bias	Not mentioned
Whitley, M. A., Walsh, D., Hayden, L., and Gould, D. (2017) [86] USA	Interpretative	Qualitative	Ad hoc questionnaire Reflective narratives Visual methods	Sample Bias Design	Deepening more
Chiva-Bartoll, Ò., Capella-Peris, C., and Pallarés-Piquer, M. (2018) [87] Spain	Interpretative	Mixed methods	Conversational techniques Reflective narratives Validated questionnaire	Design SL implementation	Not mentioned
Chiva-Bartoll, O, Pallarés-Piquer. M., Gil-Gómez, J. [58]. (2018) Spain	Interpretative	Mixed methods	Conversational techniques Reflective narratives Validated questionnaires	Instruments Sample Generalizability	Deepening more
Galvan, C., Meaney, K., and Gray, V. (2018) [53]. USA	Interpretative	Mixed methods	Specific instruments Reflective narratives Conversational techniques	Sample Generalizability	Enhance design
An, J. (2019) USA [27].	Interpretative	Qualitative	Conversational techniques Reflective narratives Visual methods	SL implementation Bias	Deepening more
Sanz, I., Calle, M. T., Aguado, R., and Garoz, I. (2019) [60]. Spain	Interpretative	Mixed methods	Validated questionnaires Narratives without reflection Reflective narratives Conversational techniques	Sample Design Generalizability	Improve the program
Capella-Peris, C., Cosgrove, M. M., Pallarès, M., and Santágueda-Villanueva, M. (2019) [51]. Spain and USA	Interpretative	Mixed methods	Validated questionnaires Reflective narratives	Sample Bias Generalizability	Deepening more Enhance design Improve the program
Chiva-Bartoll, Ó., Gil-Gómez, J., and Zorrilla-Silvestre, L. (2019) [43]. Spain	Interpretative	Mixed methods	Validated questionnaires Conversational techniques Reflective narratives	Not mentioned	Deepening more
Chiva-Bartoll, O., Salvador-Garcia, C., Capella-Peris, C., and Maravé-Vivas, M. (2019) [35]. Spain	Interpretative	Qualitative	Reflective narratives	Not mentioned	Not mentioned
Gutiérrez, D., Segovia, Y., García-López, L. M., and Fernández-Bustos, J. G. (2019) [46]. Spain	Interpretative	Mixed methods	Validated questionnaires Conversational techniques	SL implementation Design Instruments	Improve the program
Sotelino, A., Mella, I., and Rodríguez-Fernández, J. E. (2019) [74]. Spain	Interpretative	Mixed methods	Validated questionnaires Conversational techniques	Design SL implementation	Not mentioned
Maravé-Vivas, M., Gil-Gómez, J., and Chiva-Bartoll, O. (2019) [30]. Spain	Positivist	Quantitative	Validated questionnaire	Generalizability Sample Design	Deepening more
Michael, R., Webster, C. A., Nilges, L., Brian, A., Johnson, R., Carson, R., and Egan, C. A. (2019) [40]. USA	Interpretative	Qualitative	Conversational techniques Visual methods Reflective narratives	Design Sample Generalizability	Not mentioned
Pérez Pueyo, Á., Hortigüela Alcalá, D., González Calvo, G., and Fernández Río, F. J. (2019) [47]. Spain	Positivist	Quantitative	Ad hoc questionnaire	SL implementation Design	Improve the program
Ruiz-Montero, P. J., Corral-Robles, S., García-Carmona, M., and Belaire-Meliá, A. (2019) [64]. Spain	Interpretative	Qualitative	Reflective narratives	Sample Design SL implementation	Enhance design Improve program
Zorrilla-Silvestre, L., Valverde, T., Carreguí, J., and Gómez, A. (2019) [88] Spain	Positivist	Quantitative	Validated questionnaires	SL implementation	Not mentioned
Cañadas, L., and Calle-Molina, M. T. (2020) [34]. Spain	Interpretative	Qualitative	Reflective narratives	SL implementation Design	Enhance design Deepening more Improve the program
Cañadas, L., and Santos-Pastor, M. L. (2020) [57]. Spain	Positivist	Quantitative	Ad hoc questionnaires	Sample Design Generalizability Bias Instruments	Enhance design Deepening more Improve the program
Chiva-Bartoll, O., Capella-Peris, C., and Salvador-García, C. (2020) [32]. Spain	Sociocritic	Qualitative	Reflective narratives	SL implementation	Deepening more
Capella-Peris, C., Gil-Gómez, J., and Chiva-Bartoll, Ò. (2020) [89] Spain	Interpretative	Mixed methods	Validated questionnaire Narratives without reflection	Not mentioned	Improve the program Enhance design Deepening more
Chiva-Bartoll, O., Baena-Extremera, A., Hortiguela-Alcalá, D., and Ruiz-Montero, P. J. (2020) [44]. Spain	Interpretative	Mixed methods	Validated questionnaire Conversational technique	Sample SL implementation Generalizability	Enhance design Improve the program Deepening more
Chiva-Bartoll, O., Ruiz-Montero, P. J., Capella-Peris, C., and Salvador-García, C. (2020) [28]. Spain	Positivist	Quantitative	Validated questionnaires	Generalizability Sample Design	Enhance design
Lee, J., Chang, S. H., and Haegele, J. A. (2020) [29]. USA	Positivist	Quantitative	Validated questionnaires	Instruments Generalizability Sample	Deepening more
Marttinen, R., Daum, D. N., Banville, D., and Fredrick, R. N. (2020) [90] USA	Interpretative	Qualitative	Conversational techniques Reflective narratives Narratives without reflection	Not mentioned	Deepening more
Rodríguez-Costa, I., González-Rivera, M. D., Ortega, C., Llabrés-Mateu, J. M., Blanco-Morales, M., Abuín-Porras, V., and Díaz-Pulido, B. [59]. (2020) Spain	Interpretative	Qualitative	Reflective narratives Conversational techniques Visual methods	Instruments Bias Design Generalizability	Enhancing design
Rodríguez-Nogueira, Ó., Moreno-Poyato, A. R., Álvarez-Álvarez, M. J., and Pinto, A. (2020) [48]. Spain	Interpretative	Mixed methods	Validated questionnaires Reflective narratives Ad hoc questionnaire	Generalizability Sample Bias Design Instruments	Not mentioned
Ruiz-Montero, P. J., Chiva-Bartoll, O., Salvador-García, C., and González-García, C. (2020) [63]. Spain	Interpretative	Qualitative	Reflective narrative Conversational techniques	Bias SL implementation Design	Enhance design Improve the program
Santiago, J. A., Kim, M., Pasquini, E., and Roper, E. A. (2020) [55]. USA	Interpretative	Qualitative	Reflective narratives	Sample Bias Instruments SL implementation	Enhance design Deepening more
Santos-Pastor, M.L, Cañadas, L., and.; Martínez- Muñoz, L. F. (2020) [81]. Spain	Interpretative	Qualitative	Narratives without reflection Conversational techniques	Design Generalizability SL implementation	Improve the program
Valverde-Esteve, T., Chiva-Bartoll, O., Salvador-García, C., and Maravé-Vivas, M. (2020) [31]. Spain	Positivist	Quantitative	Specific instruments	Sample Design Generalizability	Enhance design Improve the program
Molina, M. T. C., Gómez, R. A., Arribas, I. S., and Rodríguez, Á. L. (2021) [41]. Spain	Interpretative	Qualitative	Reflective narratives Visual methods	Sample SL implementation	Not mentioned
Chiva-Bartoll, O., Maravé-Vivas, M., Salvador-García, C., and Valverde-Esteve, T. (2021) [52]. Spain	Interpretative	Mixed methods	Reflective narratives Conversational techniques Specific instruments	Sample Generalizability SL implementation	Improve the program
Daum, D. N., Marttinen, R., and Banville, D. (2021) [36]. USA	Interpretative	Qualitative	Reflective narratives Conversational techniques	SL implementation Design	Deepening more
García-Rico, L., Martínez-Muñoz, L. F., Santos-Pastor, M. L., and Chiva- Bartoll, O. (2021) [38]. Spain	Interpretative	Qualitative	Narratives without reflection Conversational techniques Visual methods	Design Generalizability Bias	Deepening more Enhance design
Giles Girela, F. J., García, E. R., and Cervantes, C. T. (2021) [62]. Spain	Interpretative	Qualitative	Ad hoc questionnaire Reflective narratives	Bias SL implementation	Not mentioned
Peralta, L. R., Marvell, C. L., and Cotton, W. G. (2021) [91] USA	Interpretative	Qualitative	Reflective narratives	Design Bias	Not mentioned
Santos-Pastor, M. L., Martínez-Muñoz, L. F., Garoz-Puerta, I., and García-Rico, L. (2021) [49]. Spain	Interpretative	Qualitative	Reflective narratives Ad hoc questionnaires Conversational techniques	Instruments Design	Not mentioned
